# Tamoxifen may contribute to preserve cardiac function in Duchenne muscular dystrophy

**DOI:** 10.1007/s00431-024-05670-9

**Published:** 2024-07-03

**Authors:** Bettina C. Henzi, Sebastiano A. G. Lava, Carlos Spagnuolo, Niveditha Putananickal, Birgit C. Donner, Marc Pfluger, Barbara Burkhardt, Dirk Fischer

**Affiliations:** 1 Division of Neuropediatrics and Developmental Medicine, University Children’s Hospital Basel, University of Basel, Basel, Switzerland; 2 Pediatric Cardiology Unit, Department of Pediatrics, Centre Hospitalier Universitaire Vaudois and University of Lausanne, Rue du Bugnon 46, Lausanne, 1011 Switzerland; 3 Heart Failure and Transplantation, Department of Paediatric Cardiology, Great Ormond Street Hospital, London, UK; 4 Clinical Pharmacology and Therapeutics Group, University College London, London, UK; 5 Division of Clinical Pharmacology and Toxicology, Institute of Pharmacological Sciences of Southern Switzerland, Ente Ospedaliero Cantonale, Lugano, Switzerland; 6 Division of Neuropediatrics, Development and Rehabilitation, Department of Pediatrics, Inselspital, Bern University Hospital, and University of Bern, Bern, Switzerland; 7 Pediatric Cardiology, University Children’s Hospital Basel (UKBB), University of Basel, Basel, Switzerland; 8 Department of Cardiology, Center for Congenital Heart Disease, University Hospital of Bern, Bern, Switzerland; 9 Department of Pediatric Cardiology, University Children’s Hospital Zurich, Zurich, Switzerland

**Keywords:** Duchenne muscular dystrophy, Heart failure, Cardiomyopathy, Dilated cardiomyopathy, Tamoxifen

## Abstract

Duchenne muscular dystrophy is life-limiting. Cardiomyopathy, which mostly ensues in the second decade of life, is the main cause of death. Treatment options are still limited. The TAMDMD (NCT03354039) trial assessed motor function, muscle strength and structure, laboratory biomarkers, and safety in 79 ambulant boys with genetically confirmed Duchenne muscular dystrophy, 6.5–12 years of age, receiving either daily tamoxifen 20 mg or placebo for 48 weeks. In this post-hoc analysis, available echocardiographic data of ambulant patients recruited at one study centre were retrieved and compared before and after treatment. Data from 14 patients, median 11 (interquartile range, IQR, 11–12) years of age was available. Baseline demographic characteristics were similar in participants assigned to placebo (*n* = 7) or tamoxifen (*n* = 7). Left ventricular end-diastolic diameter in the placebo group (median and IQR) was 39 (38–41) mm at baseline and 43 (38–44) mm at study end, while it was 44 (41–46) mm at baseline and 41 (37–46) mm after treatment in the tamoxifen group. Left ventricular fractional shortening in the placebo group was 35% (32–38%) before and 33% (32–36%) after treatment, while in the tamoxifen group it was 34% (33–34%) at baseline and 35% (33–35%) at study end. No safety signals were detected.

*Conclusion*: This hypothesis-generating post-hoc analysis suggests that tamoxifen over 48 weeks is well tolerated and may help preserving cardiac structure and function in Duchenne muscular dystrophy. Further studies are justified.

*ClinicalTrials.gov Identifier*: EudraCT 2017–004554–42, NCT03354039

**Table:** 

**What is known:** *• Duchenne muscular dystrophy (DMD) is life-limiting. Cardiomyopathy ensues in the second decade of life and is the main cause of death. Treatment options are still limited.* *• Tamoxifen reduced cardiac fibrosis in mice and improved cardiomyocyte function in human-induced pluripotent stem cell-derived cardiomyocytes.*
**What is new:** *• In this post-hoc analysis of the TAMDMD trial among 14 boys, median 11 years of age, treated with either tamoxifen or placebo for 48 weeks, treatment was well-tolerated.* *• A visual trend of improved left-ventricular dimensions and better systolic function preservation generates the hypothesis of a potential beneficial effect of tamoxifen in DMD cardiomyopathy.*

**What is known:**

*• Duchenne muscular dystrophy (DMD) is life-limiting. Cardiomyopathy ensues in the second decade of life and is the main cause of death. Treatment options are still limited.*

*• Tamoxifen reduced cardiac fibrosis in mice and improved cardiomyocyte function in human-induced pluripotent stem cell-derived cardiomyocytes.*

**What is new:**

*• In this post-hoc analysis of the TAMDMD trial among 14 boys, median 11 years of age, treated with either tamoxifen or placebo for 48 weeks, treatment was well-tolerated.*

*• A visual trend of improved left-ventricular dimensions and better systolic function preservation generates the hypothesis of a potential beneficial effect of tamoxifen in DMD cardiomyopathy.*

## Introduction

Duchenne muscular dystrophy (DMD) is among the most frequent hereditary muscular dystrophies and affects around 1 in 5000 boys. The disease presents with progressive muscle wasting involving skeletal and myocardial muscles. The treatment has hitherto been largely limited to symptomatic therapies and long-term corticosteroids, and life expectancy is still reduced [1–4]. Sadly, despite improvements in respiratory and physical management, the natural history of cardiac involvement in DMD has remained bleak [5–8]. Indeed, most of these patients develop progressive cardiomyopathy within the second or third decade of life [9], which is generally life-limiting [8].

In a DMD mouse model, the daily administration of tamoxifen led to an almost complete recovery of muscle strength and structure [10]. Tamoxifen, a selective oestrogen receptor modulator with both agonist and antagonist effects on different body tissues, has been a long-standing therapy for breast cancer since 1980, and broad clinical experience proves its safety and tolerability [11]. Therefore, between May 2018 and June 2021, the multicentre, randomised, double-blind, placebo-controlled phase 3 TAMDMD trial was conducted to investigate safety and efficacy of tamoxifen, repurposed in patients with DMD (EudraCT 2017–004554–42, NCT03354039) [12]. In ambulant patients, tamoxifen as an adjunct to treatment with corticosteroids did not show a significant effect on motor function as compared to placebo [13].

However, recognising the opportunity of drug repurposing for cardiovascular disease [14], and in an effort to explore the effect of tamoxifen on cardiac dimensions and function, we present here a post-hoc investigation of the ambulant TAMDMD trial cohort.

## Methods

This is a post-hoc analysis of echocardiographically estimated cardiac dimensions and systolic function among ambulant participants in the TAMDMD trial. Patients recruited at the study centre Basel, who had echocardiographic data available (either collected at this study centre or at other hospitals), were included. The design of the TAMDMD trial has been previously reported [12]. Briefly, this placebo-controlled, double-blind trial randomised 79 ambulant patients 6.5–12 years of age to either daily tamoxifen 20 mg or placebo for 48 weeks (followed by an open-label extension of additional 48 weeks). Only males with a genetically confirmed diagnosis of DMD were included. Included ambulant patients were required to be on stable glucocorticoid treatment. Children with overt heart failure were excluded [12]. The primary outcome was motor function, several secondary outcomes assessed muscle strength and structure, as well as laboratory biomarkers of DMD and tamoxifen metabolism [12]. Safety outcome parameters were obtained at each study visit. Assessed adverse events included pre-established symptoms and signs, vital signs, Tanner stage, Wells score to quantify the risk of deep vein thrombosis, ophthalmological evaluation, and several laboratory parameters [13]. The study was conducted in accordance with the Declaration of Helsinki of 1964 and its later amendments.

For this post-hoc analysis, after obtaining additional ethical approval and informed consent, the local treating physicians were contacted and asked to transmit cardiological records of visits and examinations performed before, during, and after therapy with tamoxifen or placebo. In the included patients, routine surveillance echocardiography was performed according to the recommendations of the American Society of Echocardiography by trained paediatric cardiologists [15]. Given the multicentre nature of the cardiological follow-up, both GE (equipped with S6, M5Sc, or M4S transducers) and Philips ultrasound systems (equipped with S8-3, S5-1, or X5-1 transducers) were used. Left ventricle (LV) size in M-mode and LV function as estimated by means of fractional shortening (FS) in M-mode were systematically retrieved from the echocardiographic reports and transferred to a dedicated, pseudonymized database. Before data analysis, clinical observations and echocardiographic parameters were independently checked for plausibility by a board-certified paediatric cardiologist not involved in these patients’ care.

Continuous variables are presented with non-parametric descriptive statistics, proportions as absolute numbers, and percentages. Missing data was handled by pairwise deletion. Baseline characteristics of placebo and tamoxifen groups were compared with the Mann–Whitney test (continuous variables) or Fisher exact test (proportions), as appropriate. Despite awareness of the fact that secondary analyses on non-powered samples are hypothesis-generating rather than conclusive, within-group comparisons (placebo or tamoxifen) of echocardiographic measures before and after the intervention were explored (Wilcoxon signed-rank test). Given the small sample, we opted for a graphical, explorative description of echocardiographic measurements of both groups over time. GraphPad Prism, Version 10.1.0 for Mac OS X (GraphPad Software, Boston, Massachusetts, USA) was used.

## Results

Data from 14 patients 7 to 14, median 11 years of age, with a documented diagnosis of Duchenne muscular dystrophy by mutation analysis, was available. Seven of them had been randomised to tamoxifen and seven to the placebo group. There were no significant differences in baseline demographic and clinical characteristics between treatment groups (Table 1).

**Table 1:** Demographic and clinical characteristics of study participants. Continuous variables are presented as median (interquartile range), proportions as absolute count (percentage)

	Placebo	Tamoxifen	*p* value
	(*N* = 7)	(*N* = 7)	
Characteristics			
Age [years]	12 [11–13]	11 [ 11-12]	ns
Body weight [kg]	23.6 [22.8–28.6]	29.3 [26.0–37.3]	ns
Height [cm]	118 [115–126]	128 [120–136]	ns
BMI [kg/m^2^]	18.1 [16.2–19.8]	18.6 [17.8–20.5]	ns
BSA [m^2^]	0.81 [0.80–0.85]	0.98 [0.95–1.20]	ns
Heart rate [beats/min]	97 [93–108]	89 [80–92]	ns
Systolic blood pressure [mmHg]	107 [101–119]	106 [101–110]	ns
Diastolic blood pressure [mmHg]	69 [62–77]	62 [61–65]	ns
Therapy			ns
Steroids	7	7	
ACE-inhibitor	0	1*	
Beta-blockers	0	0	
MRA	0	0	
Diuretics	0	0	

^*^lisinopril p.o. 2.5 mg/kg once daily

*ACE*, angiotensin converting enzyme; *BMI*, body mass index; *BSA*, body surface area; *MRA*, mineralocorticoid receptor antagonist; *ns*, non-significant

**Fig. 1:**
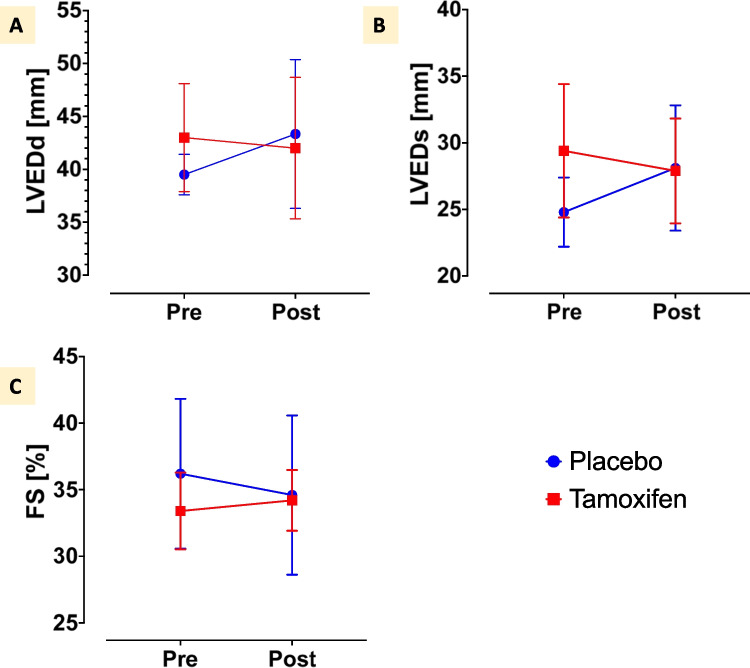
Echocardiography characteristics in the placebo (blue) and tamoxifen (red) groups. The charts depict mean (placebo group: points, tamoxifen group: squares) and standard deviation (bars) of left ventricular end-diastolic (LVEDd, panel **A**) and end-systolic (LVEDs, panel **B**) dimensions, respectively, left ventricular fractional shortening (FS, panel **C**) before (pre) and after (post) the 48-week treatment period

Echocardiographic characteristics were available at baseline for at least 7 (50%), at last available echocardiography for at least 9 (64%) participants (Tables 2 and 3). Given this low yield, a statistical comparison between the two groups over time was not possible. However, we could graphically perceive a tendency towards a better cardiac preservation in the tamoxifen group: this was reflected in a slightly decreasing left ventricular end-diastolic (LVEDd) and end-systolic (LVEDs) dimensions, as well as a stable to minimally increasing fractional shortening (FS) in the tamoxifen group, while the tendency was the opposite in the placebo group (Fig. 1), as it would have been expected as part of the natural disease course. Despite the qualitative impression of similar trends, within-group comparisons of baseline versus end-of-study echocardiographic measurements were non-significant in both groups (Tables 2 and 3). No significant differences in the occurrence of pre-defined adverse events were detected, and no patient had to stop treatment because of drug-related side effects [13].

**Table 2:** Echocardiographic characteristics of participants in the placebo group at baseline and at the last-available echocardiography

	Before	After	*p* value	No. of available data (pre/post)
LVEDd [mm]	39 [38–41]	43 [38–44]	ns	4/5
LVEDs [mm]	26 [25–26]	26 [25–31]	ns	4/5
LV-FS [%]	35 [32–38]	33 [32–36]	ns	5/5

*LVEDd*, left ventricle end-diastolic dimension; *LVEDs*, left ventricle end-systolic dimension, *LV-FS*, left ventricle fractional shortening; *ns*, non-significant

**Table 3:** Echocardiographic characteristics of participants in the tamoxifen group at baseline and at the last-available echocardiography

	Before	After	*p* value	No. of available data (pre/post)
LVEDd [mm]	44 [41–46]	41 [37–46]	ns	4/4
LVEDs [mm]	30 [27–32]	28 [25–31]	ns	3/4
LV-FS [%]	34 [33–34]	35 [33–35]	ns	5/5

*LVEDd*, left ventricle end-diastolic dimension; *LVEDs*, left ventricle end-systolic dimension; *LV-FS*, left ventricle fractional shortening; *ns*, non-significant

## Discussion

This hypothesis-generating post-hoc analysis on a small sample of young DMD boys, median 11 years of age, shows that tamoxifen over 48 weeks was well tolerated and suggests that tamoxifen might contribute to a better preservation of echocardiographically estimated cardiac dimensions and systolic function in DMD patients, so that confirmatory studies appear justified. Indeed, in front of an otherwise progressive natural disease course, a trend towards decreased left-ventricular dilation and a stabilised left-ventricular systolic function appeared to exist among children receiving tamoxifen for 48 weeks as compared to children receiving placebo. Given the small sample size of this post-hoc analysis, statistical comparisons were not meaningful and cannot be relied upon.

Nevertheless, these preliminary, exploratory results are novel and may entail important clinical implications. Indeed, cardiac disease has become the main life-limiting condition in DMD [8]. Because of the lack of DMD-specific drugs, present strategies for established DMD-related cardiomyopathy are based on treatment for adult heart failure [8], while available evidence is unfortunately still limited in paediatrics [16–20]. Improving cardiac management of DMD patients is therefore critical and urgently needed.

It is important to interpret the results of this preliminary experience and post-hoc analysis with an understanding of its inherent limitations, which are a direct consequence of its design. First, this post-hoc analysis grounded on obtainable data: the study was not designed to investigate the potential effect of 48 weeks of tamoxifen on echocardiography. Second, the available sample was small, translating into a very low power. Missing data hampered the analysis even further and mandated an exploratory, graphical approach. Within-group pre/post comparisons are reported for completeness but with full awareness that they are statistically impossible to interpret. Third, clinical symptoms and signs before and after treatment could not be compared, and NT-proBNP was not measured. Fourth, echocardiography was performed as per clinical routine in different local hospitals and collected measures were scanty (ejection fraction was not available as per the gold standard Simpson’s biplane method, diastolic function was not assessed), and the images could not be double-checked in a blind fashion by an independent paediatric cardiologist. Furthermore, *z* scores for LVEDd and LVEDs were not always documented and, weight and height not having been systematically collected at each echocardiography, they could not be reliably calculated at baseline and last available echocardiography, so that we had to rely on measurements of absolute dimensions. However, given the limited echocardiographic windows in DMD patients, fractional shortening (available in our database) has been proposed as a reliable LV systolic function measure in this population [21]. Fifth, it is meanwhile well-known that symptoms (because of reduced physical activity in DMD patients), NT-proBNP, and standard echocardiography are late markers and therefore suboptimal in investigating DMD-associated dilated cardiomyopathy. Advanced echocardiography (including regional LV motion analysis, speckle tracking, and strain analysis) and, especially, cardiac magnetic resonance imaging (assessing dimensions, function, water content, and fibrosis) are currently recommended for early detection of cardiac involvement in studies on DMD-associated cardiomyopathy [22, 23]. Sixth, tamoxifen dose was chosen based on the preliminary results of a previous open-label trial [12], but a solid dose rationale [24], based on current understanding of paediatric pharmacokinetics and comprehension of the purported cardiac effects, is still lacking. Finally, early recognition of cardiac muscle involvement, and standard institution of preventative angiotensin converting enzyme inhibitors from the age of ~ 10 years are the current mainstay of cardiac management in DMD children and adolescents [22]. Despite this, only one out of the 14 included patients was on treatment with lisinopril. Sadly, this is not surprising, and just represents one more example of the therapeutic nihilism that still characterises the current approach to paediatric heart failure [25]. This also implies that the present results might not be automatically generalised to a cohort treated according to current recommendations [22].

Despite all these limitations, the descriptive results we obtained are internally consistent, suggesting the possible existence of a real underlying biological process: LV dimensions (both end-diastolic and end-systolic) mildly worsened in the placebo, and slightly improved in the tamoxifen group, while fractional shortening (as simple but reliable systolic function measure), mildly worsened in the placebo group and minimally improved in the tamoxifen group. Furthermore, this observation is consistent with previous research, pre-clinical data, and pathophysiological considerations. In a mouse model of DMD, tamoxifen reduced cardiac fibrosis by approximately 50% [10], and arrested fibrosis processes are expected to translate into preserved relaxation, contractility, and left ventricular dimensions. Even more relevantly, on monolayers of human-induced pluripotent stem cell-derived cardiomyocytes, the active metabolite 4-hydroxytamoxifen reduced beating rate, raised beating velocity, improved calcium-handling, and prolonged viability [26].

Notably, this post-hoc analysis should be seen as hypothesis-generating [27]. In the drug development journey, we are still far from confirming any clinical effect. However, these preliminary observations suggest that efforts to define a dose rationale, followed by well-powered studies using appropriate endpoints, designed to answer the question of a possible benefit of tamoxifen on cardiac function in DMD patients, may be worth considering. The potential clinical impact on the lives of affected patients and their families might be substantial.

## Data Availability

The data presented in this study is available upon reasonable request from the corresponding author.
